# An economic evaluation of the controlled temperature chain approach for vaccine logistics: evidence from a study conducted during a meningitis A vaccine campaign in Togo

**DOI:** 10.11604/pamj.supp.2017.27.3.12087

**Published:** 2017-06-23

**Authors:** Mercy Mvundura, Patrick Lydon, Abdoulaye Gueye, Ibnou Khadim Diaw, Dadja Essoya Landoh, Bafei Toi, Anna-Lea Kahn, Debra Kristensen

**Affiliations:** 1 Devices and Tools Program, PATH, Seattle, USA; 2 Department of Immunization, Vaccines and Biologicals, World Health Organization, Geneva, Switzerland; 3 Senegal Country Program Office, PATH, Dakar, Senegal; 4 Independent consultant, Dakar, Senegal; 5 Togo Country Office, World Health Organization, Lomé, Togo; 6 Immunization Division, Ministry of Health, Lomé, Togo; 7 Devices and Tools Program, PATH, Geneva, Switzerland

**Keywords:** Costing, vaccine supply chain, controlled temperature chain (CTC), economics, meningitis A vaccine, campaign, cold chain logistics, Togo

## Abstract

**Introduction:**

A recent innovation in support of the final segment of the immunization supply chain is licensing certain vaccines for use in a controlled temperature chain (CTC), which allows excursions into ambient temperatures up to 40°C for a specific number of days immediately prior to administration. However, limited evidence exists on CTC economics to inform investments for labeling other eligible vaccines for CTC use. Using data collected during a MenAfriVac™ campaign in Togo, we estimated economic costs for vaccine logistics when using the CTC approach compared to full cold chain logistics (CCL) approach.

**Methods:**

We conducted the study in Togo’s Central Region, where two districts were using the CTC approach and two relied on a fullCCL approach during the MenAfriVac™ campaign. Data to estimate vaccine logistics costs were obtained from primary data collected using costing questionnaires and from financial cost data from campaign microplans. Costs are presented in 2014 US dollars.

**Results:**

Average logistics costs per dose were estimated at $0.026±0.032 for facilities using a CTC and $0.029±0.054 for facilities using the fullCCL approach, but the two estimates were not statistically different. However, if the facilities without refrigerators had not used a CTC but had received daily deliveries of vaccines, the average cost per dose would have increased to $0.063 (range $0.007 to $0.33), with larger logistics cost increases occurring for facilities that were far from the district.

**Conclusion:**

Using the CTC approach can reduce logistics costs for remote facilities without cold chain infrastructure, which is where CTC is designed to reduce logistical challenges of vaccine distribution.

## Introduction

Several vaccines commercialized for use in low- and middle-income countries, such as vaccines against meningitis A (MenA), hepatitis B, and cholera, can withstand higher temperatures without losing potency [1, 2]. Despite their heat stability, most are only licensed for storage and distribution at 2°C to 8°C until administration (the full cold chain logistics (CCL) approach). This poses logistical challenges in low- and middle-income countries where cold chain infrastructure is lacking [3–5] - especially in remote locations and where ambient temperatures approach 40°C. In 2012, a breakthrough in vaccine regulation occurred when a vaccine against MenA-MenAfriVac™- was licensed and labeled for use in a controlled temperature chain (CTC); that is, the vaccine can be stored and distributed at temperatures up to 40°C for a period not exceeding four days immediately prior to administration [2]. Since then, several other vaccines have been approved for use in a CTC, including the 4-valent human papillomavirus vaccine (HPV) in June 2016. Other vaccines such as the oral cholera vaccine and hepatitis B birth dose vaccine are in the process of being licensed for use in a CTC. The World Health Organization recommends the CTC approach be considered for campaigns or special vaccine delivery strategies, when possible, for CTC-licensed vaccines. The CTC approach is not currently recommended during routine vaccine delivery [6]. To date, CTC approach has only been used with the MenA vaccine; pilots for using the HPV vaccine in a CTC are being planned.

Distributing vaccines in a CTC simplifies the logistics of handling vaccines starting at the location where the CTC begins, removing the need for additional freezers to prepare icepacks, saving health worker time for logistics, and potentially increasing the geographical reach of vaccines and hence coverage [6]. A modeling study evaluated the economic benefits of CTC use [7], but field data demonstrating actual implementation cost differences and benefits of CTC use are limited. Such information could significantly facilitate country planning and decision-making with respect to CTC strategies and be used to inform continued investments in CTC, which could enable other eligible vaccines to be labeled for CTC use. As such, this study aims to provide empirical evidence on the economics of the CTC approach based on a cost analysis conducted during a MenAfriVac™ campaign held in Togo in 2014.

## Methods

### Study aims

We compared the estimated economic costs for vaccine storage and distribution when using the CTC approach to the fullCCL approach. We also modeled what the logistics costs would have been for facilities that did not have cold chain equipment if they had not used the CTC approach. In addition, we compared the closed-vial vaccine wastage rates for facilities using the CTC approach and those using the fullCCL approach.

### Study design and setting

We conducted a costing study in one of the four regions performing a MenAfriVac™ introduction campaign in Togo in 2014. The Central Region was selected as the study setting on the recommendation of the Ministry of Health, whom had been requested to select a region for the study that was representative of Togo’s supply chain challenges and would reflect the benefits of CTC use. Of the Central Region’s four districts, two (Tchaoudjo and Tchamba) used the fullCCL approach, and the other two (Sotouboua and Blitta) implemented a CTC approach. The decision on which districts used CTC and which did not was made by the Ministry of Health based on their assessment of the cold chain and logistics realities of each district and whether CTC would be beneficial. We used purposive sampling to select four health centers in each of the four districts to ensure variation of two criteria: size (the target population for the campaign) and access (the distance between the district vaccine store and each health center). The target populations for the campaign for the health centers in our sample ranged from 1,200 to 12,000, and one-way distances ranged from 0 km to approximately 200 km between the district vaccine store and the facilities. In addition, the national level and the regional level were included in the sampling as they are part of the vaccine supply chain. A total of 22 sites were included in the data collection: national level (n=1), regional level (n=1), districts (n=4), and health centers (n=16).

### Description of the cold chain infrastructure in the Central Region

A review of the microplans revealed that of the 94 health centers in the Central Region, approximately 30% did not have refrigerators for vaccine storage. Analyzing these data by district indicated that between 17% and 61% of the health centers in each district did not have refrigerators. In addition, 19% of the health centers had refrigerators that were reported as broken or out of use, and this ranged from 9% to 29% of the refrigerators in each district. Despite the lack of refrigerators in nearly half of the health centers in the region, none reported that refrigerators had been provided specifically for use during the campaign, even for the facilities that were using the full CCL approach. Instead of augmenting the cold chain equipment, the system dealt with the limited availability of cold chain equipment by having districts make daily deliveries of vaccines to the health centers without refrigerators.

### Costing data collection, sources and metrics

Quantitative data for the costing analysis were collected using structured campaign costing questionnaires adapted to the specific context of Togo. These questionnaires were used to interview health workers (immunization managers, vaccine and cold chain logisticians, drivers, and vaccinators at each level of the health system). The questionnaires used an ingredients-based costing methodology [8] to gather cost data and relied on translating the general description of the vaccine delivery strategy into the components comprising the utilization of resources—quantities and prices. Advantages of the ingredients-based costing approach include: quantities and unit costs are specified so that analyses can easily identify the most important cost drivers and their variability; scenarios can be modeled using the data collected.

Primary data were collected during two field visits. The first visit was held in October 2014 to collect data on vaccine storage and distribution for the campaign from the national vaccine warehouse and Central Region. A second field visit was conducted in December 2014 during the campaign to collect district- and service-level data.

Table 1 shows the unit costs collected for selected resources, such as health worker salaries, official per diem rates, and unit costs of energy (for data on fuel and electricity costs). Other secondary data sources, such as the national comprehensive multiyear plan for immunization [9], provided the unit costs of vehicles, while the data on unit costs of cold chain equipment were obtained from the Cold Chain Equipment Manager database [10]. The campaign monitoring data provided the number of vaccines delivered to each facility and the number of vaccine doses used at each service delivery point, and included closed-vial vaccine wastage data.

**Table 1:** Unit prices in 2014 US$ and quantities of selected resources used during the Men A campaign in Togo

Resource	Amount	Source of data
***Cost of selected cold chain equipment***		
Cold room (20 m^3^)	$46,389	[10]
TCW 3000 AC refrigerator	$3,200	[10]
RCW 50 EG refrigerator	$2,250	[10]
Sibir V110 EK refrigerator	$1,500	[10]
MF 314 freezer	$831	[10]
Electricity per kwh (US$)	$0.26	Togo
Petrol per liter (US$)	$1.38	Togo
Four-wheel drive truck (US$)	$54,586	[9]
Vehicle rental costs for vaccine delivery to Central Region (US$)	$1,926	Togo Ministry of Health. Men A Microplan, unpublished, 2014.
***Median monthly salary for health workers by job grade***		
Grade D	$208	Togo government salary scale.Unpublished.
Grade C	$304	Togo government salary scale.Unpublished.
Grade B	$454	Togo government salary scale.Unpublished.
Grade A3	$522	Togo government salary scale.Unpublished.
Grade A2	$550	Togo government salary scale.Unpublished.
Grade A1	$700	Togo government salary scale. Unpublished.
Grade Magistrate	$889	Togo government salary scale.Unpublished.
***Per diems for logistics staff and drivers (per person per day)***		
National vaccine store	$21–$38	Togo Ministry of Health. Men A Microplan, unpublished, 2014.
District logisticians	$8.51	Togo Ministry of Health. Men A Microplan, unpublished, 2014.
District drivers	$6.38	Togo Ministry of Health. Men A Microplan, unpublished, 2014.
***Number of doses of MenA vaccine delivered***		
National store	2,981,000	Togo Ministry of Health. Synthesis of MenA campaign activities, unpublished, 2014.
District stores (range)	110,350–161,160	Togo Ministry of Health. Synthesis of MenA campaign activities, unpublished, 2014.
Health centers (range)	1,400–14,580	Togo Ministry of Health. Synthesis of MenA campaign activities, unpublished, 2014.

Abbreviation: Men A, meningitis A

We analyzed the data for the costing analysis in a Microsoft Excel spreadsheet. We collected all costs in West African CFA, then converted them to 2014 US dollars using an exchange rate of US$1 to 470 CFA, the rate used in the microplans. The main outcome metric used in this analysis was the average logistics cost per dose (CPD) delivered at each level: national, district, and service delivery. At this latter level, the cost metric was calculated for CTC and the fullCCL approach.

### Types of costs and costing categories

A combination of financial and economic costs were collected and analyzed. The financial costs captured direct expenditures incurred for the campaign (such as per diems, fuel allowances, transport costs, additional cold chain or other equipment purchased specifically for the campaign), whereas the economic costs expanded the scope of the analysis by including the opportunity costs of using existing resources available from the routine immunization program that were “borrowed” for the campaign [8]. These resources indirectly contributed to the campaign (existing cold chain equipment, vehicles, and health workers involved with vaccine management). While no direct campaign expenditures were associated with these resources, their utilization was estimated and converted into an economic cost to account for all direct financial and economic costs needed to generate a full economic cost estimate [8].

The estimated costs were grouped into three main categories: (a) cold chain costs; (b) transportation costs for logistics; and (c) human resources costs for logistics, including per diems. The cold chain costs included the cost of the equipment used to store the MenA vaccine at all levels of the vaccine supply chain. This included costs for the cold rooms and refrigerators for storing vaccines, freezer rooms and freezers to make frozen icepacks, and cold boxes and vaccine carriers for storing vaccines during transport. For facilities that had cold chain equipment, the annual depreciation and energy costs were allocated to the ten-day campaign, assuming that the equipment was exclusively used for storing the MenAfriVac™ vaccines. All cold chain equipment costs (including cold boxes and vaccine carriers) were annualized using the straight-line depreciation method and assuming a five-year useful life [11].

During the campaign, health centers relied on a transportation system organized at the district level around planned delivery circuits to continually resupply vaccination posts with vaccines and frozen icepacks. The analysis included the financial costs of transportation for vaccines, which were calculated based on fuel and distances traveled, and of renting vehicles for logistics. The analysis also included the depreciation cost of using existing available vehicles as part of the national immunization program but mobilized for use during the campaign, and the cost of using private or personal vehicles, particularly at health centers. At times, additional trips were required outside of the scheduled distribution, and the costs of these trips were factored into the analysis.

Using information on local monthly salaries by staff type, the economic cost of human resources for campaign logistics was estimated for the existing staff employed to work on the national immunization program, but mobilized to work on the planning and implementation of the MenA campaign. The estimation of staff time was based on information collected via direct interviews and field observations during the campaign. Only the human resources costs for health workers and drivers with logistics duties were included in our reported estimates since our focus was on logistics costs: this included human resources time spent on pre-campaign activities associated with cold chain and logistics and the time that was spent on organizing cold boxes and vaccine carriers, conditioning icepacks, and traveling to and from vaccination sites for logistics during the campaign. The financial costs for the human resources category were based on official per diems provided to health workers for logistics activities related to the campaign.

### Costs if the CTC approach had not been used

We conducted an analysis to estimate the transport costs for vaccine delivery if sampled health centers without refrigerators had not used the CTC approach but had received daily deliveries. We compared this estimate with the estimated costs when they used the CTC approach, enabling vaccines to be stored in vaccine carriers or cold boxes at the health centers for up to four days. For the CTC approach, we assumed three deliveries would be made to each health center during the ten-day campaign. These costs show the savings from using the CTC approach in facilities that did not have refrigerators.

## Results

### Estimated logistics costs at the national and district vaccine stores

**Figure 1:**
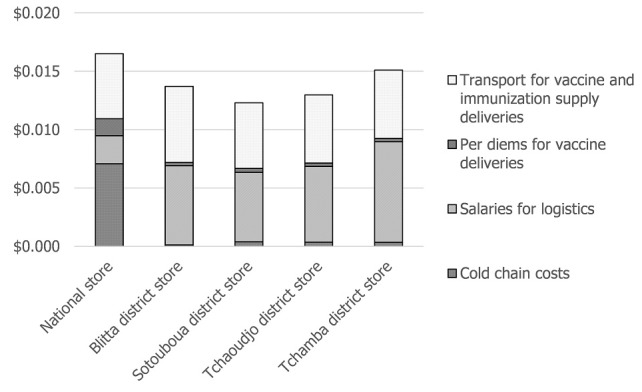
Estimated logistics costs per dose at the national and district vaccine stores

We estimated the logistics costs at the national store at $49,210 for the campaign; the largest share of the total logistics costs were for cold chain (43%) and transportation of vaccines to the districts (34%). For the cold chain costs, maintenance of equipment was the largest share of costs (90%), including maintenance of a private-sector cold room, which was rented to store routine vaccines to make space for the MenA vaccine at the national store. The estimated logistics CPD at the national store was $0.017. Figure 1 shows a logistics CPD breakdown by cost category.

At the district vaccine stores, the two largest components of logistics costs for the campaign were for human resources and transport of vaccines to the health centers (Figure 1). The transport costs at the district included one vaccine delivery trip to each health center in the district. If additional deliveries had to be made to any of the health centers in our sample, these transport costs were included under the health center costs. Cold chain costs comprised a small share of district costs (between 1% and 3%) because vaccines were stored at the district level only for the ten days of the campaign; this is not a significant economic cost given that the campaign vaccines were stored in refrigerators already in use for the routine immunization system. Total economic costs per district for the campaign ranged from approximately $1,670 to $2,200. Between 110,000 and 161,000 doses were stored in the districts, resulting in an estimated average logistics CPD of $0.014 (standard deviation of $0.001).

### Estimated logistics costs per dose at the health centers

**Figure 2:**
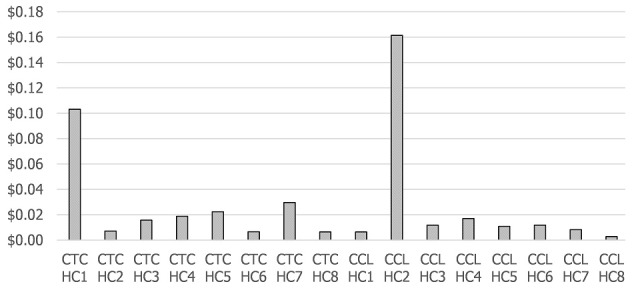
Estimated logistics costs per dose for health facilities

Figure 2 estimates the logistics CPD for each health center for the campaign. The average CPD for health centers using the CTC approach was estimated at $0.026 (standard deviation $0.032; range $0.007 to $0.103), while the average CPD for the health centers using the cold chain approach was $0.029 (standard deviation $0.054; range $0.003 to $0.161). The difference between the means for the two groups of facilities was not statistically significant. We found that health centers without refrigerators (the health centers labeled CTC HC1, CTC HC3, CTC HC4, CTC HC7, CCL HC2, and CCL HC4 in Figure 2 had higher CPDs than those with refrigerators. These higher costs were mainly driven by the transport costs for additional vaccine deliveries from the district.

**Figure 3:**
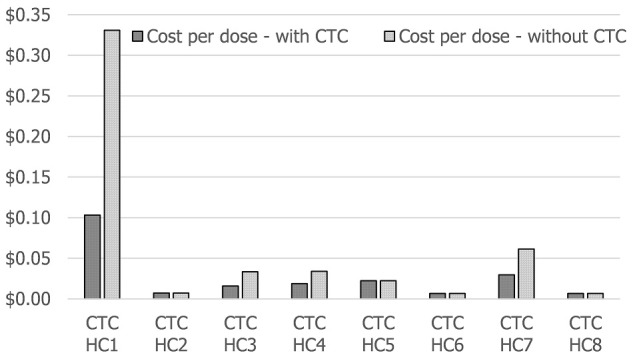
Estimated logistics costs per dose for health facilities in the CTC subsample

We modeled the transport costs for the health centers without refrigerators that were using the CTC approach to show what the costs would have been had they received daily deliveries of vaccines from the districts rather than three deliveries during the campaign. The average CPD for all the facilities using a CTC would have increased to $0.063 (range $0.003 to $0.33) per dose had they not used a CTC. The upper range is for one health facility that was located 200 km from the district and did not have a refrigerator (the health center labeled CTC HC1 in Figure 3; the CPD for this facility would have increased to $0.33 per dose if they had not used the CTC approach, compared to $0.10 per dose with a CTC.

### Estimated economic savings associated with the CTC approach

We calculated the economic savings if the MenA vaccine could be stored in a CTC from the national store to the health centers during this campaign (Table 2). The savings would have ranged from $0.03 to $0.107 per dose, with the latter estimates including the savings achieved by not purchasing the additional cold chain equipment for the campaign. These savings translate to approximately $88,000 if the cold chain costs include only the existing equipment and $319,000 if the new cold chain equipment is factored in.

**Table 2:** Estimated economic savings in 2014 US$ if the Men A vaccine had been kept in a CTC

Cost description	CPD if the existing cold chain only is considered	CPD if new cold chain equipment had been delivered for the campaign
Cold chain costs at the national store	$0.007	$0.085
Cold chain costs at the district stores	$0.0003	$0.0003
Cold chain costs at the health centers	$0.002	$0.002
Additional transport costs between the districts and health centers	$0.02	$0.02
Total CPD that could have been saved	$0.029	$0.107
Estimated economic savings if a CTC had been used from the national level to the health centers	$87,559	$318,813

Abbreviations: CPD, cost per dose; CTC, controlled temperature chain; Note: All costs are presented in 2014 US dollars

### Closed-vial wastage

The CTC approach can potentially result in higher closed-vial wastage if ambient temperatures exceed 40°C, which would cause the peak threshold indicators to reach endpoint, or if vaccines taken for CTC use remain unused after the four-day period. None of the health facilities in our sample of facilities using the CTC approach reported any vaccine wastage because of either reason. For the facilities using the fullCCL approach, closed-vial wastage can result from diluents or vaccines freezing due to exposure to ice or frozen icepacks used in a carrier during transport. None of the health centers in our fullCCL approach sample reported any closed-vial wastage because of this reason. Therefore, there was no difference in closed-vial wastage between the two approaches.

## Discussion

This study evaluated the economic benefits of using the CTC approach, focusing on the costs and savings for the logistics system during a vaccine campaign. Few previous published studies evaluated the economic benefits of thermostable vaccines [12–14]; one published study and one unpublished study explored the economic benefits of CTC use [7, 15]. We found that the economic costs for the cold chain at each level of the health system were small (<$0.01 per dose) when we considered only the costs of the existing cold chain equipment used during a campaign. This result is not unexpected because the economic costs are allocated to the campaign only for the duration of the campaign, which is a short period of time compared to the time the equipment is used to store routine immunization program vaccines.

Estimates from our study are lower than those reported by previous studies evaluating CTC use [7, 15]. There are several possible reasons for this difference. First, Togo procured $235,000 in new cold chain equipment to be used for the MenA campaign and this included 39 refrigerators and 6 freezers, but the equipment was not delivered in time for the campaign. Had these costs been included, the CPD at the national level would have been approximately $0.08 higher, if we assumed that all the costs would be allocated to the campaign. Second, the previous studies were modeled estimates of what the cost savings of implementing a CTC could be when compared with the fullCCL approach. Therefore, any comparison is difficult between a modeled estimate and a cost estimate from a setting where CTC use was actually implemented, as in our study. Third, Togo’s context is different from the countries in which the modeling studies were conducted. In Togo, the campaign relied on existing cold chain infrastructure, which was not augmented, and this meant that transportation costs were higher at the health center level, with more frequent vaccine deliveries to health centers that did not have refrigerators. In the countries modeled, additional and temporary cold chain equipment and generators were deployed in zones where there was inadequate cold chain when they were implementing the full CCL approach. As such, the cold chain cost difference between CTC and fullCCL approach areas was more pronounced in these other countries because of the costs of augmenting the cold chain.

Our analysis showed that the strongest case for CTC use is for remote health centers without cold chain equipment. The CTC approach can result in significant savings within the logistics system for such facilities. Our analysis showed that for one such health center, approximately $0.23 per dose could be saved by using a CTC rather than making daily deliveries, and there could be additional savings in human resources time dedicated to preparing icepacks.

If MenA vaccine could have been stored outside the cold chain from the national store to the vaccination point, Togo would not have had to purchase the additional equipment. The savings would have ranged from $0.03 per dose to $0.10 per dose, including the economic costs of existing cold chain equipment.

Another benefit that the CTC approach can bring is to enable potent vaccines to be available at remote locations without cold chain, and this can potentially increase coverage. Our study was not designed to test whether coverage increased between districts or health centers that were using a CTC versus those using the full CCL approach. However, we reviewed coverage data for the eight health centers in our sample that were using the CTC approach during the MenA campaign and found that for these eight facilities, there was an 8% increase in coverage during the MenAfriVac™ campaign when these sites implemented the CTC approach compared to the coverage achieved during a polio campaign held in October 2014 using the full CCL approach.

Our study also found that there was no significant difference in closed-vial wastage between the health centers using the CTC approach when compared to those using the full CCL approach. An analysis of the administrative data collected by the Togo Ministry of Health as part of the post-campaign evaluation also showed that wastage rates were not significantly different between the health facilities using the CTC approach and those using the full CCL approach.

Our study has several limitations that impacted the way the logistics system operated and may have affected our estimates. First, delays in receiving the MenAfriVac™ vaccines from the international level meant that the standard operating procedure for distributing vaccines from the national level to the regional level was not followed. Once the vaccines arrived at the national warehouse, they were dispatched directly to all districts, bypassing the regional stores. Although data were collected at the regional level to factor this level into the costing, these had to be excluded from the analysis to avoid double counting since the regional level was circumvented to expedite the distribution of vaccine to districts and avoid any delays in the start of the campaign. As a result, we may have underestimated the cold chain costs because the costs at the regional level were not included in the analysis. Similarly, transport costs may have been greater than anticipated in the microplans because of the potential inefficiencies created when the national store had to deliver vaccines to the districts: the initial plan was for the national store to deliver vaccines to the regions and have the regions deliver vaccines to the districts. Togo’s cold chain infrastructure is weak, and for remote health centers without refrigerators, the long distances (remoteness of the locations and difficult access being one of the reasons to select the CTC sites) inflated the overall cost of transport in CTC sites. Additional studies are needed to increase the evidence on the economics of the CTC approach.

## Conclusion

We found that using the CTC approach will reduce the costs of conducting a campaign in remote locations, without increasing closed-vial vaccine wastage. These findings have potential beyond reducing the logistics cost of conducting a campaign. A CTC approach allows vaccinators to extend their reach to remote areas that would otherwise be hard to reach based on the cold chain infrastructure, mitigating the risk of vaccine damage caused by inadvertent freezing as well as reducing the burden on health workers associated with the preparation of conditioned icepacks.

### What is known about this topic

- Several vaccines commercialized for use in low- and middle-income countries, such as vaccines against meningitis A (Men A), hepatitis B, and cholera, can withstand higher temperatures without losing potency;
- The cold chain infrastructure in some low- and middle-income countries is weak, posing logistical challenges for vaccine storage and transport and so any innovations that reduce the strain on the cold chain would be beneficial;
- Using the controlled temperature chain approach can simplify vaccine logistics during campaigns and facilitate outreach at locations beyond the cold chain, allowing for improved coverage and equity.

### What this study adds

- To our knowledge this is the first study that provides evidence on the economic costs and savings of using the CTC approach compared to the full cold chain logistics approach where the evidence was generated using data collected during a campaign implementing the CTC approach;
- The study findings demonstrate that using the CTC approach can reduce the costs of conducting campaigns and these savings are higher for remote locations;
- The study also finds that closed-vial vaccine wastage is not higher when using the CTC approach compared to the full CCL approach.

## Competing interests

The authors declare no competing interests.
